# New Earth-Abundant Thin Film Solar Cells Based on Chalcogenides

**DOI:** 10.3389/fchem.2019.00297

**Published:** 2019-04-30

**Authors:** Alessia Le Donne, Vanira Trifiletti, Simona Binetti

**Affiliations:** Department of Materials Science and MIBSOLAR Center, University of Milano-Bicocca, Milan, Italy

**Keywords:** earth-abundant elements, binary chalcogenides, quaternary chalcogenides, low-cost solar cells, thin film PV

## Abstract

At the end of 2017 roughly 1.8% of the worldwide electricity came from solar photovoltaics (PV), which is foreseen to have a key role in all major future energy scenarios with an installed capacity around 5 TW by 2050. Despite silicon solar cells currently rule the PV market, the extremely more versatile thin film-based devices (mainly Cu(In,Ga)Se_2_ and CdTe ones) have almost matched them in performance and present room for improvement. The low availability of some elements in the present commercially available PV technologies and the recent strong fall of silicon module price below 1 $/W_p_ focused the attention of the scientific community on cheap earth-abundant materials. In this framework, thin film solar cells based on Cu_2_ZnSnS_4_ (CZTS) and the related sulfur selenium alloy Cu_2_ZnSn(S,Se)_4_ (CZTSSe) were strongly investigated in the last 10 years. More recently, chalcogenide PV absorbers potentially able to face TW range applications better than CZTS and CZTSSe due to the higher abundance of their constituting elements are getting considerable attention. They are based on both MY_2_ (where *M* = Fe, Cu, Sn and *Y* = S and/or Se) and Cu_2_XSnY_4_ (where *X* = Fe, Mn, Ni, Ba, Co, Cd and *Y* = S and/or Se) chalcogenides. In this work, an extensive review of emerging earth-abundant thin film solar cells based on both MY_2_ and Cu_2_XSnY_4_ species is given, along with some considerations on the abundance and annual production of their constituting elements.

## Introduction

In the last decades, the fast increase of the global energy demand and the progressive run-up in the world oil price, along with the growing global pollution strongly pointed out the need of an affordable and sustainable clean energy supply. As a matter of fact, the main energy sources since the Industrial Revolution were coal, oil, and natural gas, whose combustion is primarily blamed on the CO_2_ emission into the Earth's atmosphere responsible for many global climate changes. In order to face the 28 TW global energy demand foreseen for 2050 (Hoffert et al., 1998) without affecting the environment, a wider employment of renewable energies is therefore mandatory.

**Figure 1:**
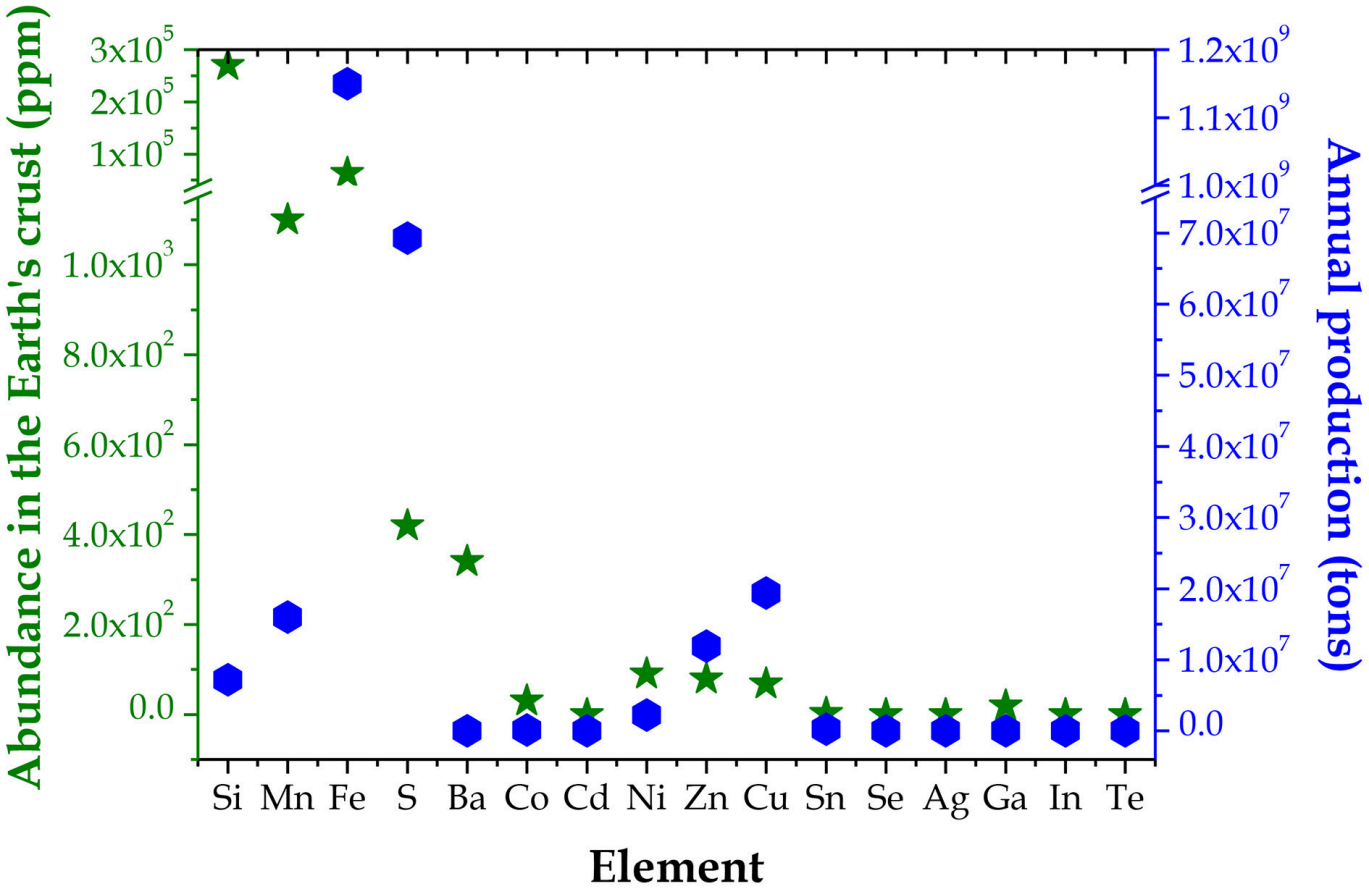
Abundance in the Earth's crust (left green y axis) and annual production (right blue y axis) of several elements (USGS; WebElements).

In the last 15 years, all renewable energies grew strongly in many end-use sectors (power, heating/cooling, and transport), in particular, at the end of 2017, roughly 1.8% of the worldwide electricity came from solar photovoltaics (PV), which was the main source of new power capacity in many countries, including China, India, Japan, and United States. Globally, the newly installed capacity of solar PV in 2017 was around 98 GW (about 29% more than the record additions in 2016), which raised the cumulative total up to 402 GW (REN21, 2018). In order to meet a noticeable portion of the global energy demand foreseen for 2050 (Hoffert et al., 1998), the installed capacity of solar PV will have to expand at least to 5 TW. However, almost all of the present commercially available PV technologies suffer from material or resource constraints that will likely limit their future role in TW scale applications. Currently, crystalline-Si (c-Si) based devices rule the solar PV market, accounting for about 94% of the total annual production vs. 6% for all alternative PV technologies (namely, thin-film CdTe, thin-film Cu(In,Ga)Se_2_ (CIGS), and thin-film Si) (Fraunhofer ISE, 2017). The above mentioned limitations include the relevant energy amount required to fabricate c-Si solar cells and the low availability of one or more elements present in CdTe (i.e., Te), CIGS (i.e., In and Ga), c-Si and thin-film Si (i.e., Ag used as contact) PV devices (Tao et al., 2011) (see also Figure 1, USGS; WebElements). To overcome this problem, in the last 10 years thin films based on earth abundant elements were strongly investigated as PV absorbers, in particular, Cu_2_ZnSnS_4_ (CZTS) and the related sulfur selenium alloy Cu_2_ZnSn(S,Se)_4_ (CZTSSe). CZTS and CZTSSe, in the stable crystalline kesterite form (tetragonal, space group I4, unit cell: *a* = 5.427 Å, *c* = 10.871 Å; *Z* = 2), have direct bandgap around 1.5 and 1.1 eV, respectively, and high absorption coefficient (over 10^4^ cm^−1^) (Ito, 2014; Liu et al., 2016). They both showed promising performance, namely 9.2% record efficiency for CZTS (Sun et al., 2016) and 12.6% for CZTSSe (Wang et al., 2014). Although there has been considerable progress in the performance of kesterite solar cells, further improvements are needed to achieve the high efficiency required for the practical application of these types of devices. First of all, the defects inside the material must be reduced to limit the recombination processes and the formation of band tail. But although several processes have been tested, such as the increase in doping and various treatments after deposition, still the defectiveness and the presence of deleterious secondary phases have not been sufficiently reduced. In fact, during the growth of CZTS films, given the complexity of the phase diagram, numerous secondary phases and intrinsic defects are produced. Complex and not yet fully understood are the relationships between growth process, chemical composition, and transport or recombination properties of photo-generated carriers. Moreover, the architecture of the most commonly used device is the same used for the realization of CIGS devices, although it is not actually optimized for kesterite (Ito, 2014). For example, the CdS/CZTS interface does not have an optimal band bending: an alternative layer would improve the charge transport and free the process of CZTS device realization from the use of cadmium (Santoni et al., 2013; Kumar et al., 2015). To overcome this problem, alternative buffer layers have been proposed, for instance ZnCdS (Congiu et al., 2018) and a Zn_1−x_Cd_x_S film that is able to optimize the conduction band offset, therefore diminishing the charge recombination (Sun et al., 2016).

As a matter of fact, a cheap PV technology requires both low-cost raw materials and low-cost production processes. As far as the cost of raw materials is concerned, elements with high abundance in the Earth's crust are good candidates for TW range PV applications, however both mining and processing of the minerals must be cheap as well. As an example, Ga is relatively abundant in the upper continental crust (roughly comparable to Cu, Ni, Co, and Sn) (WebElements), nevertheless producing 100 tons of Ga would imply in the best scenario the handling of 2,000,000 tons of bauxite (Tao, 2014), which makes Ga expensive. Getting to the heart of the present matter, Zn is widely known as element under serious threat of extinction in the next 100 years (Harland et al., 2013), due to the combination of limited abundance in the earth crust, limited annual production and huge employment in many applications. As a consequence, in a TW scale PV scenario also CZTS will suffer from material or resource constraints, even if to a lower extent with respect to the present commercial PV devices. Therefore, CXTS absorbers based on significantly abundant elements must be developed and used along with CZTS to face the need of a 5 TW PV installed capacity by 2050. As shown in Figure 1, Fe, Mn, Ni, Ba, Co, and Cd are good and/or possible alternatives to Zn in Cu_2_XSn(S, Se)_4_ materials as well as Cu, Sn, and Fe are good candidates for M(S, Se)_2_ chalcogenides, since they all show a convenient abundance to annual production ratio (USGS; WebElements). As far as the production cost is concerned, the development of cheap and easily scalable PV absorber layers strongly depends on growth techniques, which are usually classified as either vacuum or non-vacuum. About the vacuum methods, the sputtering technique is suitable to up-scaling the system in terms of active area and efficiency, while maintaining a good control of the deposition rate, while evaporation is one of the meritorious techniques to grow absorber layers without impurities. The non-vacuum methodologies, on the other hand, are lately attracting more and more attention in order to lower the production cost.

In this work, an extensive review of emerging earth-abundant thin film solar cells based on both MY_2_ and Cu_2_XSnY_4_ (where *M* = Fe, Cu, Sn; *X* = Fe, Mn, Ni, Ba, Co, Cd; *Y* = S and/or Se) species is given, along with some considerations on the abundance and annual production of their constituting elements.

## Earth-Abundant Elements for Chalcogenide PV Absorbers

### Iron

Iron provides top abundance to annual production ratio, that is 63000 ppm vs. 1.15 × 10^9^ tons (USGS). Iron in fact makes up about 5% of the Earth's crust, being therefore the fourth most abundant element after oxygen, silicon and aluminum (WebElements). The iron is found in Nature mainly as iron oxide minerals such as siderite (FeCO_3_), magnetite (Fe_3_O_4_), and hematite (Fe_2_O_3_). Moreover, the lower mantle of the Earth mainly consists of iron based compounds, that is ferropericlase (Mg,Fe)O and silicate perovskite (Mg,Fe)SiO_3_ (Murakami et al., 2012). Industrial iron production uses iron ores, mainly magnetite and hematite, and it involves a carbothermic reaction to reduce the sources to the metal (Remus et al., 2013).

### Manganese

Manganese is the 12th most abundant element, making up about 0.1% (1100 ppm) of the Earth's crust (WebElements). This value coupled with the high annual production of 1.6 × 10^7^ tons (USGS) makes manganese a cheap element. Manganese occurs in the Earth's crust principally as pyrolusite (MnO_2_), rhodochrosite (MnCO_3_), psilomelane (Ba,H_2_O)_2_Mn_5_O_10_, and braunite ((SiO_4_)Mn_6_O_8_), the most important ore being pyrolusite (Bhattacharyya et al., 1984). In the 70s, it was estimated that the ocean floor had 500 billion tons of Mn nodules, however they were never exploited due to the absence of economically viable extraction methods (United Nations Ocean Economics Technology Office, 1978). Many manganese deposits are spread around the world, about 80% of them being located in South Africa. Other important producers are Ukraine, Australia, India, China, Gabon, and Brazil (Corathers, 2009). Pure Mn is produced by leaching manganese ore with sulfuric acid and subsequently employing an electrochemical process (Zhang and Cheng, 2007).

### Barium

Barium is the 14th most abundant element in the Earth's crust making up a 0.0425% of it (340 ppm) (WebElements). The main commercial source of barium is barite (BaSO_4_), a mineral with deposits in many parts of the world, principally in England, Romania, and Russian Federation (Kresse et al., 2007). The mined ores are washed, crushed, and separated from quartz. Barite with at least 95% purity undergoes a series of chemical reactions to produce pure Ba. No precise estimations of the annual Ba production are available, which has few industrial applications. However, it is known that about 8 × 10^6^ barite tons are annually produced (Kuck, 2012).

### Sulfur

Sulfur is the 17th most common element in the Earth's crust (420 ppm) and generally the fifth most common on Earth (WebElements). Although it is also present on Earth in its pure, native form, sulfur usually occurs as sulfide and sulfate minerals, such as pyrite (iron sulfide), galena (lead sulfide), sphalerite (zinc sulfide), cinnabar (mercury sulfide), stibnite (antimony sulfide), alunite (potassium aluminum sulfate), gypsum (calcium sulfate), and barite (barium sulfate) (Anthony et al., 1990). Presently, elemental sulfur is mainly obtained as a byproduct of purifying natural gas and fossil fuels (Eow, 2002). Around 69'300'000 tons of sulfur are produced annually worldwide (USGS), which allows to fulfill the large sulfur demand related to its application in many fields.

### Nickel

Nickel is the 24th most abundant element in the Earth's crust (80 ppm) (WebElements). The most important sources of nickel are limonite (FeO(OH)·*n*H_2_O), pentlandite ((Fe,Ni)_9_S_8_), and garnierite (H_2_O(Mg,Ni)_3_Si_4_O_10_(OH)_2_) (Anthony et al., 1990). The world's largest nickel producers are Philippines, Indonesia, Russian Federation, Canada, and Australia (Kuck, 2012). Nickel is produced using ore by extractive metallurgy, employing conventional roasting and reduction processes which generate a metal with purity over 75% (Nickel Institute). Around 2'250'000 tons of nickel are produced annually worldwide (USGS), however Ni is used in a plethora of applications: about 68% of world production for stainless steel, 9% for corrosion-resistant nickel plating, 10% for nickel and copper -based alloys, 7% for alloy steels, 3% in foundries, and 4% in other applications, including the fast-growing battery sector (Nickel Institute).

### Elements With Limited Abundance in the Earth's Crust

Zinc, copper, cobalt, tin, cadmium, silver and selenium are, respectively, the 25th (79 ppm), 26th (68 ppm), 32nd (30 ppm), 49th (2.2 ppm), 64th (0.15 ppm), 65th (0.08 ppm), and 67th (0.05 ppm) most abundant elements in the Earth's crust, with annual production around 12'000'000 tons, 19'400'000 tons, 123'000 tons, 280'000 tons, 23'000 tons, 27'000 tons, and 2'200 tons, respectively (USGS; WebElements). The abundance to annual production ratios of zinc, cobalt, cadmium, silver, and selenium and their large use in many sectors make them less suitable than the previously described elements for TW range PV applications. Conversely, the significant annual production of copper and tin, which may be properly recycled as well (Scott et al., 1997; Kasper et al., 2011), relieves their relatively limited abundance and accounts for the extensive research activity on Cu and Sn based alloys.

## Emerging Earth-Abundant Chalcogenide PV Absorbers

### Binary Chalcogenides

#### FeS_2_, FeSe, and FeSe_2_

**Figure 2:**
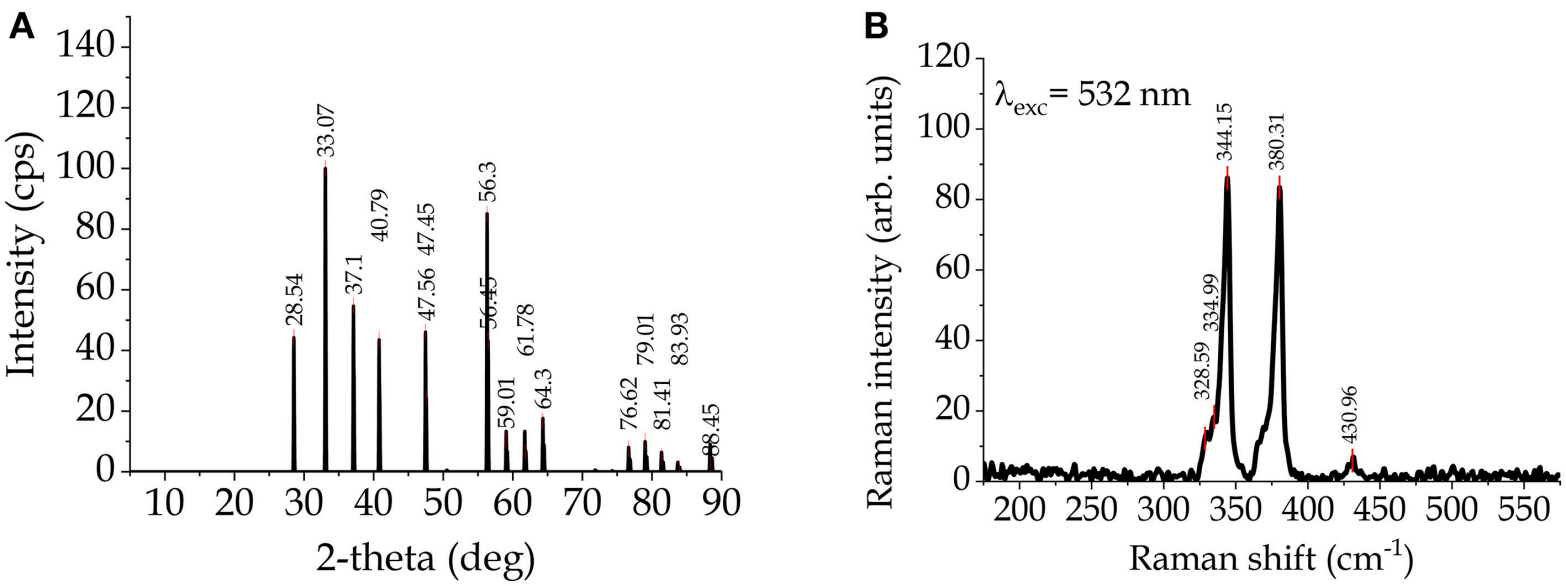
**(A)** XRD pattern and **(B)** Raman spectrum of FeS_2_.

Iron sulfide, FeS_2_ (iron(II) disulfide), is a mineral called pyrite, or iron pyrite, and also called fool's gold for its metallic luster and pale brass-yellow hue, that makes it looks like gold. Due to its abundance in nature and its low toxicity, FeS_2_ is a possible PV material. Its extraction cost is so low that a pyrite-based solar cell with only 4% efficiency could be as economical as a monocrystalline silicon solar cell with 20% efficiency (Wadia et al., 2009). Pyrite has a very high absorption coefficient (~5 × 10^5^ cm^−1^) and an energy bandgap (E_g_ ~ 0.95 eV) suitable for converting PV energy. XRD pattern and Raman spectrum are reported in Figures 2A,B, respectively^1^. Various methods for the synthesis of thin films of pyrite have been adopted, such as Fe_2_O_3_ sulphuration, sputtering, spray pyrolysis (SP), vacuum evaporation (VE), chemical bath deposition (CBD), molecular beam epitaxy (MBE), electrochemical deposition, and colloidal NC synthesis (Bi et al., 2011; Puthussery et al., 2011; Morrish et al., 2012; Bai et al., 2013; Prabukanthan et al., 2017).

FeS_2_ has a high potential in the large-scale production of PV modules, but for a long time, it was hard to obtain well workable thin-film devices that use pyrite as an absorber (Bi et al., 2011). Indeed, a significant PV efficiency value (8.39%) (Huang et al., 2015) was obtained by using the FeS_2_ not as absorber material, but as a counter electrode in a DSC solar cell (Kilic and Turkdogan, 2017). The limiting factors are the high dark current, due to phase impurities, and a high density of acceptor surface states (Cabán-Acevedo et al., 2014). Moreover, FeS_2_ is affected by thermal instability (Yu et al., 2011). Lately, Prabukanthan et al. (2017) synthesized FeS_2_ thin films by electrochemical deposition at 70°C and reported efficiency of 1.98% for a solar cell with architecture ITO/FeS_2_/ZnSe/Au (PV parameters in Table 1). With the aim of enhancing the photoresponse and stabilize the structure, they have introduced 3 mole% Co^2+^ as a dopant, reaching a remarkable efficiency of 5.42%. They suggest that substituting Co^2+^ for Fe^2+^ makes the bandgap decrease, improving the charge separations at the interface with ZnSe. X-ray diffraction and Raman spectra confirmed that doped and undoped FeS_2_ thin films have a cubic pyrite structure; the atomic force microscopy revealed that the 3 mole% of Co^2+^ doped FeS_2_ thin film exhibits a smoother surface and lower roughness values (Prabukanthan et al., 2017).

**Table 1:** Conductivity, performance, grain size (large grains LG or nanocrystals NC), concomitant reference and publication year for all of the PV devices reported in this work.

**Material**	**Conductivity**	**Efficiency (%)**	**V_**oc**_ (V)**	**J_**sc**_ (mA/cm^**2**^)**	**FF (%)**	**Grain size**	**References**
FeS_2_	p-type	1.98	0.57	6.55	53	NC	Prabukanthan et al., 2017
Co^2+^ (3% mol) doped FeS_2_	p-type	5.42	0.83	10.71	61	NC	Prabukanthan et al., 2017
Cu_2_S	p-type	9.15	0.52	19.3	71.4	LG	Bragagnolo et al., 1980
Cu_2_S	p-type	1.6	0.57	5.625	49.4	NC	Wu et al., 2008
SnS	p-type	4.4	0.372	20.2	58	LG	Sinsermsuksakul et al., 2014
heterostructure SnS/SnS_2_	p-type/n-type	0.51	0.12	10.87	39	LG	Gedi et al., 2016
heterostructure SnS/SnS_2_	p-type/n-type	1.4	0.53	5.7	46.5	NC	Degrauw et al., 2017
SnSe	p-type	1.42	0.299	11.6	41	LG	Minnam Reddy et al., 2018
CFTS	p-type	2.95	0.61	9.3	52	NC	Chatterjee and Pal, 2017
CFTS	p-type	0.11	0.13	3.25	26.6	LG	Meng et al., 2016
CMTS	p-type	0.49	0.308	4.7	33.9	LG	Chen et al., 2015b
CMTS	p-type	0.38	0.359	2.95	35.8	LG	Chen et al., 2016a
CMTS	p-type	0.73	0.381	4.95	38.6	LG	Prabhakar et al., 2016
CMTS	p-type	0.33	0.23	4	36.3	LG	Marchionna et al., 2017
CMTS	p-type	0.83	0.35	5.8	40	LG	Le Donne et al., 2017
CBTS	p-type	1.54	0.699	4.1	53.5	LG	Shin et al., 2016
CBTS	p-type	5.2	0.611	17.4	48.9	LG	Shin et al., 2017
CBTSSe	p-type	1.57	0.613	6.78	37.7	LG	Ge et al., 2016
CCdTS	p-type	2.7	0.513	12.7	42	LG	Timmo et al., 2013
CCdTSSe	p-type	3.1	0.356	20.89	41.6	LG	Zhao et al., 2015
AZTSe	n-type	5	0.5	22	49	LG	Gershon et al., 2016a
(Ag_x_Cu_1−x_)_2_ZnSnSe_4_	p-type	10.2	0.423	38.4	62.9	LG	Gershon et al., 2016b

Iron selenides can be found in two stoichiometric phases, FeSe and FeSe_2_. This last one, also called ferroselite, has been investigated as an electrode material in tandem PV; it is a p-type semiconductor material with a 1.0 eV bandgap (Qurashi, 2014).

#### CuS, Cu_2_S, and Cu_2_Se

Cuprous chalcogenide films, CuS, Cu_2_S, Cu_2_Se, are p-type semiconductors. They can be considered ideal materials for use in low-cost and non-toxic solar cells. Cu_2_S, for example, is an indirect gap semiconductor with a bandgap of 1.21 eV, which is optimal for use as a light absorber (Liu et al., 2003). Since the early 80s, thin-film solar cells based on a CdS/Cu_2_S junction were produced, reaching efficiencies of 9.15%, with open circuit voltage (V_oc_) of 516 mV, short circuit current density (J_sc_) equals to 19.3 mA/cm^2^ and fill factor (FF) of 71.4% under a sunlight intensity of 87.9 mW/cm^2^ (Bragagnolo et al., 1980). Subsequently, the research on these devices was abandoned due to the diffusion of Cu^+^ in CdS, to the high electron-hole recombination, and to the possibile coexistence of mixed phases ranging from CuS_2_, that has a metallic conduction (Munson et al., 1967), to Cu_2_S, which could give to the material a quasi-metallic behavior (Moitra and Deb, 1983; Niemegeers and Burgelman, 1986; Page et al., 2009). Nowadays, Cu_2_S is successfully employed as sensitizer in DSCs: Mousavi-Kamazani et al. (2016) employed Cu_2_S quantum dots as a barrier layer in DSCs, showing a considerable improvement in the efficiency of about 37%. In thin film architecture, the last efforts are devoted to stabilizing the solar cells: Wu et al. (2008) presented the synthesis of colloidal Cu_2_S nanocrystals and realized a solution-processed solar cell, coupled with CdS nanorods, with 1.6% efficiency and 4 months stability. Cu_x_S shows at room temperature five stable phases, ranging from the more stable structure chalcocite (*x* = 2), to the defective structure djurleite (*x* = 1.94), digenite (*x* = 1.80), anilite (*x* = 1.75), and covellite (*x* = 1.00); mixed phases has been observed in the intermediate compositions. Congiu et al. (Congiu et al., 2016), have indeed proposed an ink-based method for realize Cu_2−x_S counter electrode for DCS, showing high stability using ferrocene as redox shuttle. Various grown methods are reported: CBD (Loferski et al., 1979), successive ionic layer adsorption and reaction (SILAR) (Mani et al., 2014), MBE (Gautier et al., 1998), VE (Nair et al., 1998), solid state reaction (Sanchez Ranjel et al., 2015), SP (Sabah et al., 2015), hydrothermal method (Patil et al., 2018), atomic layer deposition (ALD), and chemical vapor deposition (CVD) (Ye et al., 2015). CuS is 2D metal chalcogenide (Karthick Kannan et al., 2015) mostly employed as a hole transport material in solar cells, rather than as a light absorber (Lei et al., 2014; Rao et al., 2016), and thanks to the high catalytic activity, proved to guarantee high performing DSCs (Li et al., 2014; Zhang et al., 2016a,b).

Copper selenides exist in many phases and structural forms, such as CuSe (klockmannite), Cu_2_Se_x_, CuSe_2_ (marcasite), α-Cu_2_Se (bellidoite), Cu_3_Se_2_ (umagnite), Cu_5_Se_4_ (athabaskite), Cu_7_Se_4_, and in isometric form as Cu_2−x_Se (berzelianite). They may also have different crystallographic forms (monoclinic, cubic, tetragonal, hexagonal, etc.). Copper selenides are p-type semiconductors, with both direct and indirect bandgap (Petrović et al., 2017), which may be potentially employed as PV absorbers. CuSe at room temperature has a hexagonal structure and undergoes an orthorhombic transition at 48°C and a hexagonal transition at 120°C. At higher temperatures, CuSe decomposes into Cu_2−x_Se and selenium. Cu_2−x_Se, at room temperature, has a cubic structure with faces centered with 0.15 ≤ × ≤ 0.2 and; when *x* = 0.2, it shows a direct bandgap of 2. 2 eV and an indirect bandwidth of 1.4 eV (Qurashi, 2014).

#### SnS and SnSe

**Figure 3:**
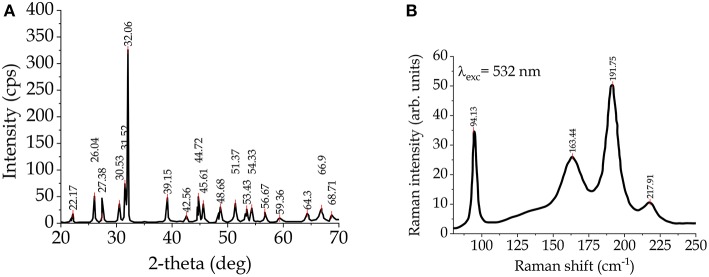
**(A)** XRD pattern and **(B)** Raman spectrum of SnS.

Tin can form sulfides with different Sn/S ratio, such as: SnS_2_, Sn_2_S_3_, Sn_3_S_4_, Sn_4_S_5_, SnS (Jiang and Ozin, 1998). The most investigated are tin(II) sulfide (SnS), with a distorted GeS structure and tin(IV) sulfide (SnS_2_) with a PbI_2_ structure (Rimmington et al., 1972). Tin(IV) sulfide can be found in 70 polytype structures, with a hexagonal close-packed structure and different c parameters (Pałosz et al., 1990). Tin monosulphide (SnS) is a mineral called herzenbergite, and the synthesized SnS is a p-type semiconductor material, as the tin vacancies generate acceptor levels (Thangaraju and Kaliannan, 2000). Figure 3 shows the XRD pattern (a) and the Raman spectrum (b) for single-crystal SnS (Raadik et al., 2013).

When then tin to sulfur ratio is SnS_0.8_, it has a bandgap of 1.16 eV (Price et al., 1999), which is like that of silicon, but with a higher optical absorption coefficient. Although the maximum theoretical efficiency of SnS thin-film solar cells is 32%, the maximum efficiency is 4.4%, reported by Sinsermsuksakul et al. (2014). They deposited, by ALD, SnS films (400 nm), using alternating doses of tin precursor vapor and a gas mixture of 4% H_2_S in N_2_, to enlarge the grains and reduce the recombination at the boundaries. Additionally, they engineered the n-type Zn(O,S) buffer layer (30 nm), reducing the sulfur and adding nitrogen as a dopant, to improve the rectifying quality of the p-n junction, and introduced a 1 nm thick SnO_2_, to enhance the quality of the SnS/Zn(O,S) interface (Sinsermsuksakul et al., 2014). The low solar cells performance can be due also to phase impurity and off-stoichiometry (Sinsermsuksakul et al., 2014). Kim and coworkers (Kim et al., 2018) investigated the presence of Sn-S polytypes (SnS, SnS_2_, Sn_2_S_3_) and their effect on the solar cells performance. SnS requires high sulfurization temperature, 500°C, meanwhile, the transition to SnS_2_ and Sn_2_S_3_ happens between 150 and 300°C (Banu et al., 2017). Unfortunately, SnS_2_ shows n-type conductivity and its presence affects the open circuit voltage. Sn_2_S_3_ has an energy gap of 1.09 eV, like the of SnS one, but a phase mixture can influence the carrier transport, generating a type II junction between Sn_2_S_3_ and SnS (Burton et al., 2013). Kim et al. (2018) did not verify the presence of Sn_2_S_3_, but they found out that the SnS_2_ phase can be predominant on the thin film surface and in the shallow bulk area. Therefore, another key point is the selection of an adequate buffer layer for the SnS absorber: lately, many researchers are focused on the realization of SnS/SnS_2_ p-n junction, with the aim to improve the SnS/buffer interface. Firstly, Sanchez-Juarez et al. (2005) fabricated an SnS/SnS_2_ thin film hetero-junction by plasma-enhanced chemical vapor deposition, verifying the PV effect. More recently, Gedi et al. (2016) fabricated SnS/SnS_2_ solar cells by CBD, the efficiency of 0.51%, proving that the junction is a type-II heterostructure. Degrauw et al. (2017) grown nanowire arrays of SnS/SnS_2_ heterojunction, by chemical vapor transport (CVT) catalyzed by Cu particles, and fabricated devices with the efficiency of 1.4 %.

Tin and selenium form tin(II) selenide (SnSe), also called stannous selenide, which is a p-type semiconductor, with both an indirect bandgap at 0.90 eV and a direct bandgap at 1.30 eV (Lefebvre et al., 1998). It is receiving increasing attention for potential applications in low-cost PV, as it is an easy processable two-dimensional layered material (Wang et al., 2016; Jeong et al., 2017; Razykov et al., 2018; Ul Haq et al., 2018). fabricated SnSe thin films by chemical molecular beam deposition, using synthesized polycrystalline SnSe as precursors: XRD analysis proves that SnSe films can be grown in the orthorhombic crystalline structure. The realized films displays a direct optical bandgap of 1.1–1.2 eV and a remarkable high absorption coefficient of about 10^5^ cm^−1^. The authors have proven that the physical properties of SnSe thin films are strongly affected by the growth conditions, and that a proper develop of the deposition methodology can lead to a material suitable for thin film solar cells. Lately Minnam Reddy et al. (2018), have fabricated a SnSe solar cells a power conversation efficiency of 1.42%.

### Quaternary Chalcogenides

#### Cu_2_FeSnS_4_

As mentioned above, iron shows top abundance to annual production ratio, therefore p-type Cu_2_FeSnS_4_ (CFTS) is a very good candidate as cheap chalcogenide PV absorber layer. In addition, CFTS shows bandgap (1.28–1.50 eV) and absorption coefficient (>10^4^ cm^−1^) suitable for PV applications.

**Figure 4:**
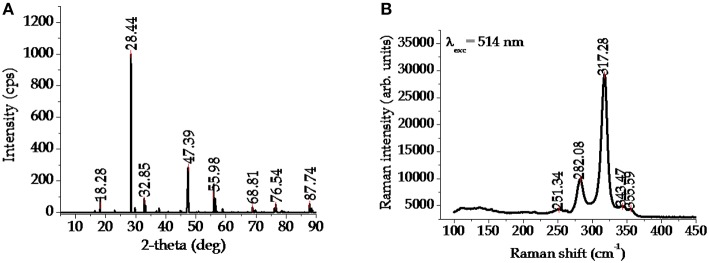
**(A)** XRD pattern and **(B)** Raman spectrum of typical stannite CFTS.

The crystal structure of CFTS, which is a lustrous dark gray mineral, is basically stannite, however early X-ray diffraction (XRD) studies demonstrated that the tetragonal stannite phase I (−42 m) is stable in the temperature range between 420 and 500°C (Quintero et al., 1999). At higher temperature, cubic polymorphous modifications with disordered sphalerite-like structure I (−43 m) were also observed (Evstigneeva and Kabalov, 2001). XRD pattern and Raman spectrum (widely used for the identification of possible secondary phases in the fabricated films) of typical stannite CFTS^2^ are depicted in Figures 4A,B, respectively.

So far, various methods have been reported for the preparation of CFTS absorber layers (Guan et al., 2014; Kevin et al., 2015; Khadka and Kim, 2015; Meng et al., 2015a, 2016; Chatterjee and Pal, 2017; Chen et al., 2017; Miao et al., 2017). Most of them are based on non-vacuum techniques, since they are simple, low-cost, often efficient and do not require sophisticated deposition set-up. Very few efficiency values were reported up to now for CFTS based PV devices, the best one being recently obtained by Chatterjee and Pal (2017) through SILAR method. SILAR is a low temperature non-vacuum technique, which offers a good balance between fabrication cost and phase purity, while being easily scalable for large area depositions. The as-formed CFTS thin films prepared in Chatterjee and Pal (2017) showed stannite structure with high crystallinity and a lower limit of crystallite size around 10 nm, as confirmed by XRD, Scanning Electron Microscopy (SEM), and cross-sectional SEM. The optical bandgap calculated through the Tauc's plot obtained from transmittance measurements was 1.5 eV, in agreement with the literature. Several n-type semiconductors were considered to complete the heterojunction, all of them obtained by SILAR as well. The best conversion efficiency of 2.95% was achieved with promising reproducibility in CFTS/Bi_2_S_3_ heterojunctions (Chatterjee and Pal, 2017). Meng et al. (2015a, 2016) also reported PV device efficiencies, obtained by solar cells based on CFTS prepared by sulfurization of the sputtered metal precursors. They showed that CFTS thin films sulfurized under fast heating rate around 40°C/min were S-poor and showed a bilayer structure with many micro-grains at the bottom of the CFTS thin film. By reducing the heating rate to 20°C/min, CFTS isolated grains were formed and the in general the grain size increases, most probably since a lower heating rate gives enough time and energy to favor the precursor crystallization and alloying. The best sputtering based CFTS solar cell prepared in Meng et al. (2016) showed a 0.11% efficiency (V_oc_ = 129 mV, J_sc_ = 3.25 mA/cm^2^, FF = 26.6%). Guan et al. (2014) reported for the first time on the preparation of CFTS thin films by the SILAR method, obtaining CFTS layers with large agglomeration of rod-shaped grains, a bandgap of 1.22 eV and an absorption coefficient higher than 10^4^ cm^−1^. Khadka and Kim (Khadka and Kim, 2015) employed electrostatic field assisted spray pyrolysis followed by sulfurization to produce stannite structured CFTS and CFTSe. Both the structural quality (i.e., crystalline texture and grain size) and the carrier mobility of CFTS/CFTSe thin films improved after the sulfur addition. Kevin et al. (2015) reported on CFTS, CFTSe, and CFTSSe thin films deposited by Aerosol Assisted Chemical Vapor Deposition (AACVD) at 350°C using mixtures of molecular precursors. More recently, CFTS thin films were prepared by doctor blade deposition of oxides (CuO, Fe_2_O_3_, and SnO_2_) on glass followed by sulfurization (Chen et al., 2017) and by electrochemical deposition followed by annealing at 500–550°C (Miao et al., 2017).

Incidentally, beside its role as low-cost photo-absorber layer in thin film solar cells, CFTS was recently considered both as counter electrodes and as cheaper alternative to platinum (Pt) in dye sensitized solar cells. An extensive review on these topics is reported in Vanalakara et al. (2018).

#### Cu_2_MnSnS_4_

As mentioned above, Mn provides relevant abundance to annual production ratio, therefore p-type Cu_2_MnSnS_4_ (CMTS) is also a good candidate as cheap chalcogenide PV absorber layer. CMTS was investigated above all as single crystal (Podsiadlo et al., 2015) or nanocrystal (Cui et al., 2012; Liang et al., 2012) for the diluted magnetic semiconductor characteristic, while, in the last few years, some papers reported on CMTS thin films for PV applications (Chen et al., 2015a,b, 2016a; Wang et al., 2015; Prabhakar et al., 2016; Le Donne et al., 2017; Marchionna et al., 2017; Yu et al., 2017). Wang et al. (2015) obtained stannite CMTS thin films by sulfurization of different precursor (Cu,Sn)S/MnS and (Cu,Sn,Mn)S films, all deposited on glass by chemical methods. The surface morphologies basically consisted of small and irregular crystalline grains (size around 18–22 nm) with a few voids. The optical bandgap values ranged between 1.15 and 1.26 eV, while the occurrence of a p-type conductivity was confirmed. Chen et al. (2015a) reported on the synthesis and the properties of stannite CMTS thin films grown by direct liquid coating and by annealing in nitrogen atmosphere, which were employed in Chen et al. (2015b) as photo-absorbers in solar cells achieving 0.49% of maximum efficiency. Some of the same authors showed also that CMTS thin films can be prepared by direct liquid coating and combining the annealing in nitrogen atmosphere and the post-sulfurization in sulfur vapors, reporting a 0.38% efficiency (Chen et al., 2016a). The same group recently investigated CMTS thin films grown by sulfurization of electrodeposited Cu-Sn/Mn metal precursors (Yu et al., 2017), which are a promising starting point for the preparation of CMTS layers by a simple, environmentally friendly and low-cost method. Prabhakar et al. (2016) presented a study on thin film solar cells based on stannite CMTS and Cu_2_MnSn(S,Se)_4_ (CMTSSe) layers, fabricated by spray pyrolysis using water as solvent. Proof-of-concept PV devices with structure Mo/CMTS/CdS/TCO provided the best performance (i.e., 0.73% efficiency) when CMTS layers were doped with Na in the form of NaCl during the spray process. Na in fact is widely known to improve grain growth and reduce non-radiative recombination both in CIGS and CZTS thin films. Through a careful electrical analysis (Hall measurements on exfoliated films and J-V curves under illumination of the PV devices), a very high carrier density in the CMTS/CMTSSe layers was identified as responsible for the low V_oc_ and FF values.

**Figure 5:**
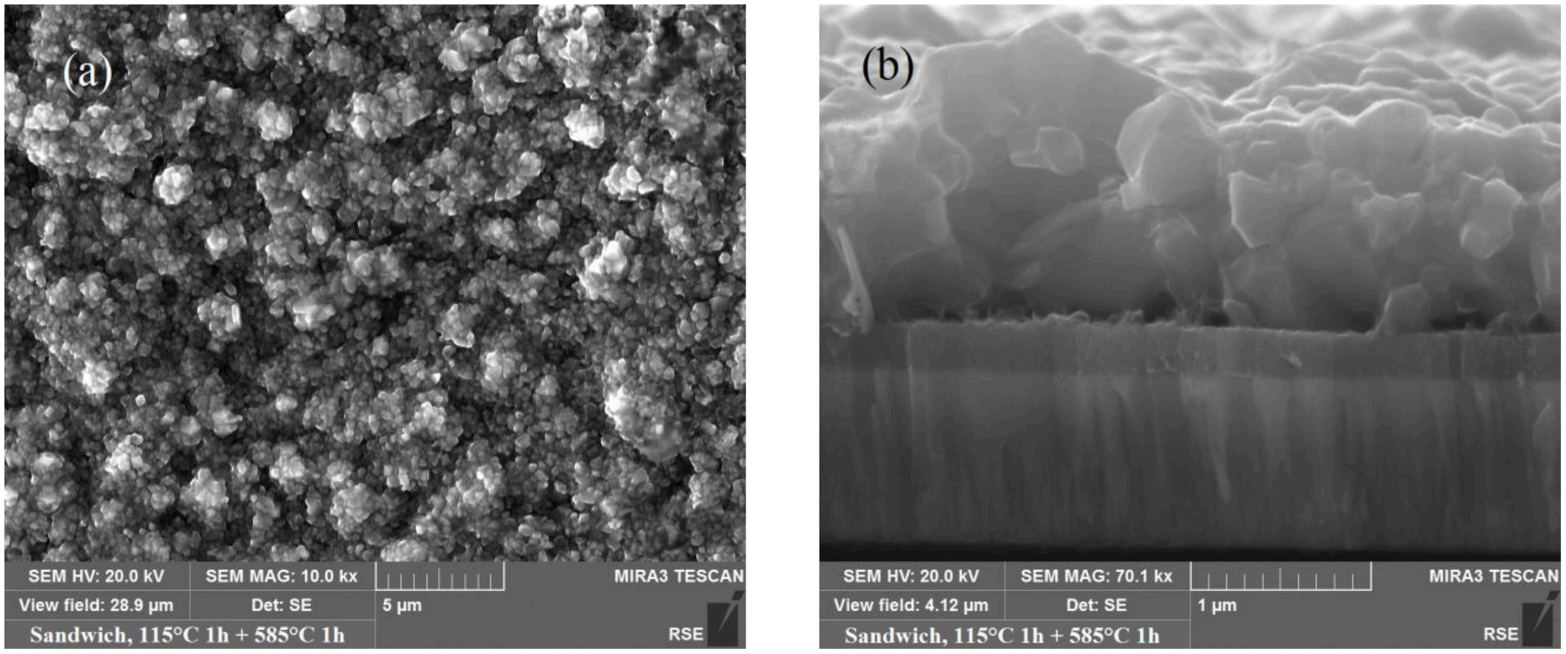
**(a)** SEM image of CMTS samples grown by sulfurization at 115 + 585°C; **(b)** cross sectional SEM image of a CMTS sample grown by sulfurization at 115 + 585°C [Reprinted from (Marchionna et al., 2017) with copyright permission from Elsevier].

Some of the present authors reported on CMTS PV absorber synthesized by a two-step process: firstly the metal precursor stacks have been deposited by thermal evaporation, and then they have been annealed in nontoxic sulfur vapors (Le Donne et al., 2017; Marchionna et al., 2017). Of the many possible stoichiometries, Cu-poor/Mn-rich CMTS films with Mn/Sn ratio around 1 were chosen to avoid the arise of both highly conductive (e.g., Cu_2−x_S) and insulating (e.g., MnS) secondary phases. In (Marchionna et al., 2017), an extensive characterization of both CMTS and any possible secondary phases by SEM, Energy Dispersive Spectroscopy, Raman, XRD, and Photoluminescence (PL) was presented. Large grain size (see Figure 5), direct band suitable for PV applications and high absorption coefficient have been reported for Cu-poor/Mn-rich samples synthesized by sulfurization with temperatures between 500 and 585°C, starting the annealing process at 115°C to trigger the alloy formation. Limited electrical performance were reached (efficiency 0.33%, V_oc_ = 226 mV, J_sc_ = 4 mA/cm^2^, FF 36.3%) (Marchionna et al., 2017), mainly due to the presence of Cu_7.38(11)_Mn_4_Sn_12_S_32_, a spinel-type insulating secondary phase.

Cu-poor/Mn-rich CMTS layers with better homogeneity of the metal ratios were presented in Le Donne et al. (2017), reached through an enhanced control of the precursor evaporation rate; PV devices showed improved performance compared to (Marchionna et al., 2017): efficiency 0.5 vs, 0.33%, V_oc_ = 302 vs, 226 mV, J_sc_ = 4.6 vs. 4 mA/cm^2^, FF 36 vs. 36.3%. Considering the beneficial consequences of low temperature post-deposition thermal treatments on kesterite based solar cells (Neuschitzer et al., 2015; Jiang et al., 2016), the effect of annealing temperature ranging from 200 to 275°C on the same CMTS devices was investigated, too. The best annealing in air at 225°C for 40 min led to a significant reduction of recombination losses without a strong increase of CdS absorption, allowing for a remarkable improvement of CMTS solar cell performance (J_sc_ = 5.8 mA/cm^2^, FF 40%, V_oc_ = 354 mV, efficiency 0.83%) (Le Donne et al., 2017).

#### Cu_2_BaSnS_4_/Cu_2_BaSn(S,Se)_4_

As it is known in the literature, V_oc_ in CZTS/CZTSSe is mainly limited by the band tailing related to cation-cation antisite disorder and by the following potential fluctuations (Gokmen et al., 2013). The main antisite defects, responsible of the antisite disorder, are copper-on-zinc (Cu_Zn_) and zinc-on-copper (Zn_Cu_) (Chen et al., 2013). Furthermore, Sn multivalency could induce deep levels in the bandgap, which in turn could cause non-radiative recombination, when Sn^2+^ occupies a Zn^2+^ site (Chen et al., 2013). Thanks to the distinct electronic properties exhibited by Cu, Sn and Ba, the formation of cation–cation antisite defects in Cu_2_BaSnS_4_ (CBTS)/Cu_2_BaSn(S,Se)_4_ (CBTSSe) has been proven to be difficult (Xiao et al., 2017). Therefore, in addition to the good abundance to annual production ratio provided by Ba, CBTS/CBTSSe could potentially lead to better optoelectronic properties than CZTS/CZTSSe. Up to now, very few works reported on CBTS/CBTSSe, which mainly deal with vacuum deposition methods (Ge et al., 2016; Shin et al., 2016, 2017). Shin et al. investigated Cu_2_BaSnSe_x_S_4−x_ films with different Se contents prepared by co-sputtering followed by sulfurization/selenization (Shin et al., 2016). Of the different examined stoichiometries, combined experimental and theoretical analyses showed that Cu_2_BaSnSe_x_S_4−x_ thin films with 0 < x ≤ 3 compositions are isostructural to CBTS with space group P3_1_ and display a tunable bandgap in the 1.6–2 eV range, which well fits the optimal values for being employed both in single junction (i.e., 1–1.6 eV) and top solar cell in multijunction (i.e., 1.7–2.0 eV) PV devices. Furthermore, optical absorption, External Quantum Efficiency and PL data confirmed the expected absence of band tailing in CBTS. Last but not least, prototype CBTS-based thin-film solar cells with 1.54% average efficiency have been prepared and tested (V_oc_ = 699 mV, J_sc_ = 4.1 mA/cm^2^, FF = 53.5%) (Shin et al., 2016). Some of the same authors obtained further improvements by combining the bandgap tuning related to the introduction of Se with a post-deposition annealing in air, producing a CBTSSe-based PV device with 5.2% efficiency (Shin et al., 2017). Ge et al. (2016) studied polycrystalline Cu_2_BaSn(Se_0.83_S_0.17_)_4_ (CBTSSe) thin films grown on fluorine tin oxide (FTO) by co-sputtering of a sulfide precursor followed by selenization. In contrast to the XRD results reported in (Shin et al., 2016), these layers (Ge et al., 2016) are isostructural to CBTSe with space group Ama2. Conversely, they exhibit, as expected, an optical bandgap around 1.85 eV, absorption coefficient higher than 10^4^ cm^−1^, and p-type conductivity. A proof-of-concept PV device with FTO/BCTSSe/CdS/ZnO/AZO structure showed 1.57% efficiency (V_oc_ = 613 mV, J_sc_ = 6.78 mA/cm^2^, FF = 37.7%) (Ge et al., 2016).

#### Cu_2_NiSnS_4_

Theoretical calculations claim that the substitution of Zn with Ni in CZTS could reduce the optical bandgap and potentially enhance electrical conductivity (Ghosh et al., 2014). These attractive features along with the sufficient abundance to annual production ratio provided by Ni make Cu_2_NiSnS_4_ (CNTS) worth to be considered for TW range PV applications. Currently, few works on CNTS are present in the literature, most of them reporting on thin films prepared by non-vacuum techniques, namely electrodeposition followed by sulfurization (Chen et al., 2016b; Yang et al., 2016), spray sandwich method (Dridi et al., 2017; Bitri et al., 2018) and direct solution coating followed by sulfurization (Mokurala et al., 2017). As expected, the layer morphology was generally found to be strongly dependent on the substrate, the larger grain size being obtained on SLG due to the higher diffusion of Na from the substrate. Hall measurements showed that after sulfurization an increase in mobility and a decrease in resistivity occur (Mokurala et al., 2017), which were attributed to the passivation of defects (mainly V_S_) and to the enhanced crystallization and in the CNTS thin films. The high absorption coefficient (>10^4^ cm^−1^) and the suitable optical bandgaps (i.e., 1.29–1.50 eV) generally obtained, along with some preliminary low resistivity values [≅ 0.14 Ωcm (Mokurala et al., 2017)], suggest that cubic p-type CNTS thin films are promising for PV applications.

#### Cu_2_CoSnX_4_, Cu_2_CdSnX_4_, and Ag Related Quaternary Alloys (X = S and/or Se)

Among the chalcogenide thin film absorbers based on less abundant elements (i.e., Co, Cd, and Ag), most of the studies reported in the literature deals with Cu_2_CdSnS_4_ (CCdTS)/Cu_2_CdSn(S,Se)_4_ (CCdTSSe). In fact, despite the low abundance to annual production ratio provided by cadmium, the replacement of Zn by the bigger Cd atom leads to a reduced presence of detrimental Zn_Cu_ antisite defects, which could enhance the conductivity (Hussain et al., 2016). Furthermore, the CCdTS bandgap is nearer than the CZTS one to the optimal value calculated by Shockley and Queisser (i.e., 1.34 eV) (Smets et al., 2016), which could potentially lead to more efficient PV devices. CCdTS, which more stable crystalline form is stannite, shows p-type conductivity related to the presence of Cu vacancies (V_Cu_) and/or Cu_Cd_ antisites (Meng et al., 2015b). So far, CCdTS/CCdTSSe was mainly investigated in the form of nanocrystal (Fan et al., 2011; Ramasamy et al., 2013), while few works reported on the deposition of CCdTS/CCdTSSe thin films (Timmo et al., 2013; Nie et al., 2015; Zhao et al., 2015; Henry et al., 2016; Xu et al., 2016; Rouchdi et al., 2017) and in particular on CCdTS/CCdTSSe based PV devices (Timmo et al., 2013; Zhao et al., 2015). Among the latter, Timmo et al. prepared CCdTS monograin powders with 1.4 eV bandgap from molten CdI_2_ or KI as flux materials sealed in quartz ampoules at 610°C, reaching a 2.7% efficiency (Timmo et al., 2013). Zhao et al. (2015) reported on stannite CCdTSSe thin films prepared by a chemical solution approach, which showed large densely packed grains and suitable bandgap around 1 eV. Solar cell prototypes based on them showed a 3.1% efficiency. However, it should be remarked that Cd as well as many Cd based compound are toxic, making CCdTS/CCdTSSe not fully compatible with a green energy supply.

Finally, few literature works explored Cu_2_CoSnS_4_ and Ag related quaternary alloys as thin film PV absorbers, namely (Krishnaiah et al., 2015; Gershon et al., 2016a,b; Ghosh et al., 2016). Particularly attractive results were obtained for Ag_2_ZnSnSe_4_ (AZTSe) (Gershon et al., 2016a) and for (Ag_x_Cu_1−x_)_2_ZnSnSe_4_ (Gershon et al., 2016b), which both showed efficiencies much higher than those reported in most of the previous paragraphs (see Table 1). Most probably, the reason is that the introduction of Ag in CZTSe was demonstrated to strongly reduce the band-tailing effect responsible for the V_oc_ loss in kesterite thin film solar cells, which was discussed above in the CBTS section. Theoretical calculations (Chagarov et al., 2016) showed in fact that the formation energy of I-II antisite in AZTSe is significantly higher than in CZTSSe, which reduces the I-II antisite density by at least one order of magnitude. The band-tailing reduction is particularly relevant for pure-Ag materials (i.e., AZTSe), however Gershon et al. (2016b) showed that introducing only 10% Ag in CZTSe allows to obtain a remarkable 10.2% efficiency. Despite a further optimization could potentially improve the performance of (Ag_x_Cu_1−x_)_2_ZnSnSe_4_ based solar cells (Gershon et al., 2016b), silver is a further element under serious threat of extinction in the next 100 years (Harland et al., 2013), so most probably they will have a marginal role in the PV TW challenge.

## Conclusion and Prospects

In this work, an extensive review of emerging sulfide/selenide materials with abundant and non-toxic elements is given. We are aware that the success of such emerging chalcogenide materials in the PV market will depend on many variables. Economic ones, including costs in $/W_p_ and added value for both consumers and architects, and mainly scientific ones. As discussed here, although in the last few years the performance of emerging chalcogenide solar cells was significantly progressing, further improvements are needed to achieve the efficiency required for the practical use. In fact, of the many absorbers reviewed here, very few provided efficiencies over 5%, as clearly summarized in Table 1. Some points were identified as critical: (a) the band-tailing effect responsible for the V_oc_ loss in kesterite thin film solar cells has to be reduced. Up to now, this goal was encouragingly achieved in CBTS and mainly in (Ag_x_Cu_1−x_)_2_ZnSnSe_4_, however silver is under serious threat of extinction in the next 100 years, so further solutions must be explored; (b) the recombination has to be reduced, for example, by passivating the defects at the grain boundaries of the photo-absorber and at the photo-absorber/buffer interface; (c) the chemical composition and the manufacturing methods must be designed to avoid secondary phases and to manage the bulk defects; (d) the architecture of the device must be adapted to the characteristics of the absorber material. As we have reviewed, the research in the field is addressing these issues but is in a preliminary phase and many efforts are necessary to reach the goal. However, the technical and environmental profile of each new discussed material as well as the easy way of production are very promising. In fact, the recent advances in emerging solutions-based PV research have opened new paths, which could soon lead to some of these limitations being overcome. Moreover, several materials have been tested to optimize the device architecture and improve the interfaces. Lately, more and more research teams from different backgrounds are joining the efforts to make thin film photovoltaics based on chalcogenides available for commercialization. Because the limits of the discussed materials are many-sided, involving issues for chemistry, physics and engineering, in order to push ahead the research on new earth-abundant thin film solar cells based on chalcogenides a more interdisciplinary research approach should be promoted. The final aim is to deliver versatile and efficient solar cells with clean and sustainable materials and that can hold a key role in energy production in the develop of that applications, such as building integration PV, outdoor recreational, and low-power consumer electronics, where the conventional solar materials cannot be applied.

## Author Contributions

AL and VT made substantial contributions to the design of the work, to the reference acquisition, analysis or interpretation of data for the work and to the writing. SB made substantial contributions to the design of the work, critical analysis or interpretation of data for the work, revising it critically, provide approval for publication of the content.

### Conflict of Interest Statement

The authors declare that the research was conducted in the absence of any commercial or financial relationships that could be construed as a potential conflict of interest.

## Footnotes

^1^RRUFF database. Available online at: http://rruff.info/chem=FeS/notchem=all/display=default/R100166

^2^RRUFF database. Available online at: http://rruff.info/chem=Cu%20Fe%20Sn%20S/display=default/R050187
